# Calcium Peroxide‐Based Hydrogels Enable Biphasic Release of Hydrogen Peroxide for Infected Wound Healing

**DOI:** 10.1002/advs.202404813

**Published:** 2024-09-03

**Authors:** Ying Huang, Zi Fu, Han Wang, Zeyang Liu, Mengqi Gao, Yanran Luo, Meng Zhang, Jing Wang, Dalong Ni

**Affiliations:** ^1^ Department of Orthopaedics Shanghai Key Laboratory for Prevention and Treatment of Bone and Joint Diseases Shanghai Institute of Traumatology and Orthopaedics Ruijin Hospital Shanghai Jiao Tong University School of Medicine Shanghai 200025 P. R. China; ^2^ Department of Emergency Ruijin Hospital Shanghai Jiao Tong University School of Medicine Shanghai 200025 P. R. China; ^3^ State Key Laboratory of Oncology in South China Collaborative Innovation Center for Cancer Medicine Guangdong Key Laboratory of Nasopharyngeal Carcinoma Diagnosis and Therapy Sun Yat‐sen University Cancer Center Guangzhou 510060 P. R. China; ^4^ Department of Radiology Shanghai Fourth People's Hospital School of Medicine Tongji University Shanghai 200025 P. R. China

**Keywords:** antibacterial treatment, calcium peroxide, hydrogels, nanomedicine, wound healing

## Abstract

Wound infection is a major factor affecting the speed and quality of wound healing. While hydrogen peroxide (H_2_O_2_) is recognized for its antibacterial capacity and facilitation of wound healing, its administration requires careful dosage differentiation. Inappropriately matched dosages can protract the healing of infected wounds. Herein, a calcium peroxide‐based hydrogel (CPO‐Alg hydrogel) is fabricated to enable a biphasic tapered release of H_2_O_2_, ensuring robust initial antimicrobial activity followed by sustained promotion of cellular proliferation of wound healing. The design of the hydrogel allowed for the calcium peroxide nanoparticles (CPO NPs) being in two spatial niches within the gel framework. When applied to infectious wounds, CPO NPs with weak constraints are promptly released out of the gel, penetrating into infected regions to serve as antibacterial agents that eliminate bacteria and biofilms at high concentrations. Conversely, the entrapped CPO NPs structurally integrated into the gel remain confined, thus gradually degrading and allowing a mild release of H_2_O_2_ through hydrolysis in a moist environment that contributes to the cell growth in the later stage. The CPO‐Alg hydrogel represents an innovative and practical solution for the antimicrobial protection of chronic wounds, offering promising prospects for advancing wound healing.

## Graphical abstract

A new nanoparticulate network hydrogel is developed by employing CPO NPs as building units to cross‐link with alginate macromolecules to form the hydrogel matrix. The CPO NPs could exist in the gel framework with two different forms. Such unique structural design enables a biphasic tapered release of H_2_O_2_. The nanoparticulate network hydrogel represents an innovative solution for the antimicrobial protection of chronic wounds.

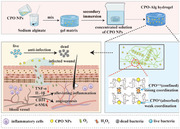

## 1 Introduction

Chronic wound management has emerged as a profound challenge to healthcare systems and imposes considerable economic burdens. Approximately 1%–2% of the world's population are expected to experience chronic wounds in their lifetime, with the annual costs for treatment exceeding $31 billion.^[^ ^1^ ^]^ Several factors can delay the wound healing process, including infection, aging, and chronic metabolic disorders. Among these, infection particularly stands as the critical element affecting healing efficacy and quality, which can potentially develop into sepsis, multiple organ failure, and in severe cases of fatality.^[^ ^2^ ^]^ Hence, infection control is pivotal in the recuperation of chronic wounds. While antibiotic therapy has become the principal modality for infection management, growing studies have highlighted a concurrent escalation in antimicrobial resistance within bacterial, which significantly diminish the effectiveness of antibiotics.^[^ ^3^ ^]^ Alternative antimicrobial strategies, such as those based on quaternary ammonium salts^[^ ^4^ ^]^ and metal ions,^[^ ^5^ ^]^ pose risks of environmental and metabolic toxicity.^[^ ^6^ ^]^ Hence, the development of novel and effective antimicrobial strategy is urgently required.

Hydrogen peroxide (H_2_O_2_), as an antimicrobial reagent, has been widely engaged in the biomedical industry,^[^ ^7^ ^]^ particularly in wound cleaning, where it eradicates pathogens through local reactive oxygen species generation and oxidative burst.^[^ ^8^ ^]^ Compared to strategies utilizing antibiotics and metal ions (e.g., Ag, Cu), H_2_O_2_ demonstrates superior broad‐spectrum antimicrobial efficacy and a reduced potential for inducing bacterial resistance. However, the inherent unfavorable properties of H_2_O_2_, including its swift breakdown, transient efficacy, biochemical reactivity, and dosage limitation, have hindered its broad application in vivo.^[^ ^9^ ^]^

Fortunately, recent progress has witnessed the emergence of calcium peroxide nanoparticles (CPO NPs), which have effectively overcome the encountered limitations associated with H_2_O_2_.^[^ ^10^ ^]^ For CPO NPs, each nanoparticle serves as a storage unit that confines H_2_O_2_ within the nanocrystal matrix, thus securing the active site of H_2_O_2_ until the external stimulus prompts release. The sustained release capabilities of these NPs for H_2_O_2_ have been demonstrated, alongside their effectiveness in infection control and tissue regeneration facilitation.^[^ ^11^ ^]^ Nonetheless, current research approaches have predominantly employed the encapsulation of CPO NPs within nanocarriers or hydrogel vehicles as prodrugs.^[^ ^12^ ^]^ The application of CPO NPs directly to wounds still presents potential risks, such as the over‐accumulation of H_2_O_2_,^[^ ^8b^ ^]^ which could incite unwarranted inflammatory responses. More importantly, treating infected wounds requires a vigorous initial oxidative response for antimicrobial success, followed by a moderated H_2_O_2_ dispersion to foster the healing process, which necessitates a highly precise release mechanism to cater to the varying stages of wound treatment.^[^ ^13^ ^]^

**Scheme 1:**
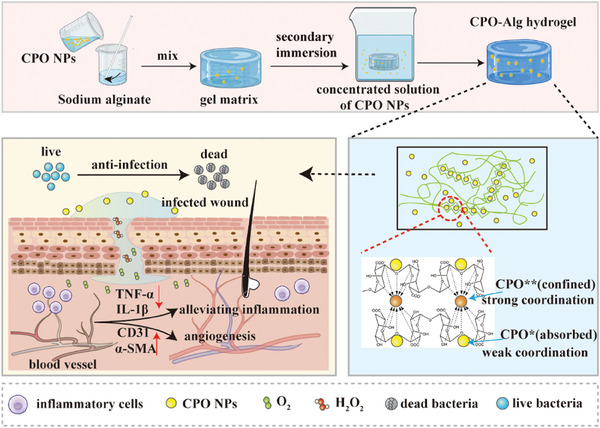
Schematic diagram of the preparation of CPO‐Alg hydrogel and their application for infected wound healing. CPO‐Alg hydrogels were prepared through a two‐step process. The two‐step fabrication of CPO‐Alg hydrogels facilitates the coexistence of CPO NPs with two distinct coordination states in the gel structure, allowing a tapered release of H_2_O_2_.

To address these challenges, in this study, a CPO NPs‐based hydrogel was developed to provide effective antimicrobial properties and promote chronic wound healing (**Scheme** **1**). The hydrogels employed CPO NPs as building units to cross‐link with alginate (Alg) macromolecules to create a primary gel matrix. Subsequent exposure to dense CPO NP solutions led to the formation of the final CPO‐Alg hydrogel. The two‐step fabrication of CPO‐Alg hydrogel endowed the coexistence of NPs in two distinct spatial arrangements within the gel framework: one segment of NPs adsorbed throughout the interspatial voids of the hydrogel, while the remainder served as pivotal network junctions reinforcing the gel architecture integrity. Consequently, hydrogels underwent a two‐stage release dynamic. The adsorbed CPO NPs initially dispersed externally and established a potent antimicrobial microenvironment. Subsequently, the immobilized CPO NPs anchored within the gel matrix nodes degraded through gradual water erosion in a milder manner, thereby effectuating a prolonged liberation of H_2_O_2_ conducive to wounds healing. The unique structural design effectively enabled a tapered release mechanism of H_2_O_2_, ensuring ample antimicrobial activity during phase I of wound repair, and subsequently encouraged cellular proliferation in phase II–IV of wound repair by a markedly reduced H_2_O_2_ accumulation. Furthermore, the hydrogel possessed excellent biodegradability and self‐absorbability because all degradation products of the hydrogel are naturally occurring substances within the living organism, further ensuring its safety and biocompatibility. This calcium peroxide‐based antibacterial hydrogel provides an innovative and practical solution for the antimicrobial protection of chronic wounds, offering promising prospects for future wound healing research.

## 2 Results and Discussion

### 2.1 Preparation and Characterization of CPO NPs and CPO‐Alg Gel

CPO NPs were prepared through a modified wet‐chemistry method in methanol‐water mixture at room temperature.^[^ ^7a^ ^]^ X‐ray diffraction (XRD) characterization confirmed the successful synthesis of CPO NPs, as each diffraction peak of the sample agreed exactly with the calcium peroxide standard pattern (PDF#03‐0865) as well as the theoretical predictions by Zhao et.al (Figure S1, Supporting Information).^[^ ^14^ ^]^ In addition, Raman spectra of the NPs validate the presence of O─O bonds (Figure S2, Supporting Information). The CPO NPs obtained were well dispersed and had excellent crystallinity, as demonstrated by Transmission Electron Microscopy (TEM) (Figure S3, Supporting Information), and the average hydrodynamic diameter of the CPO NPs was ≈93 nm as determined by Dynamic Light Scattering (DLS) (Figure S4, Supporting Information). From the high‐resolution TEM (HRTEM), the lattice spacing was calculated to be 0.240 nm. Furthermore, elemental mapping displayed a homogeneous distribution of O and Ca elements in the CPO NPs (Figure S5, Supporting Information). In addition, the CPO NPs were positively charged with the zeta potential value of +25.8 mV (Figure S6, Supporting Information). All these results demonstrated the successful synthesis of CPO NPs.

**Figure 1:**
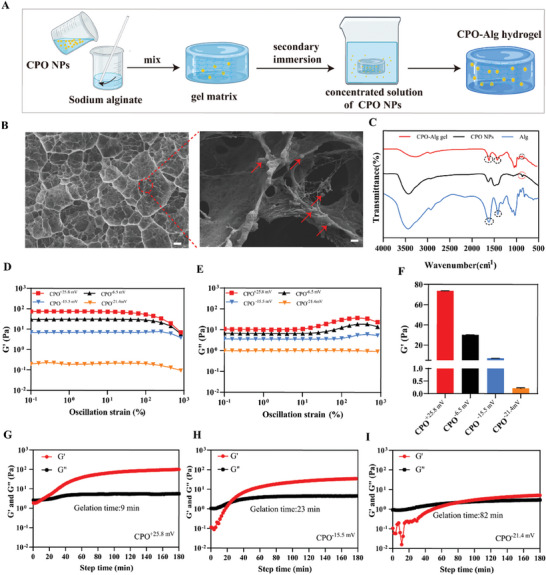
Preparation and cross‐linking mechanism of CPO‐Alg gel. A) Schematic illustration of the preparation process of CPO‐Alg hydrogel. B) SEM images of CPO‐Alg hydrogel. Scale bar, 3 µm (left), 300 nm (enlarged). C) FT‐IR spectra of CPO NPs (black line), Alg (blue line), and CPO‐Alg hydrogel (red line). D) Storage modulus (G′) value of CPO‐Alg gel matrix formed by different surface charges of CPO NPs in the strain‐sweep test. E) Loss modulus (G″) value of CPO‐Alg gel matrix formed by different surface charges of CPO NPs in the strain‐sweep test. F) G′ value of CPO‐Alg gel matrix formed by different surface charges of CPO NPs at strain of 1%. G, H, I) G′ and G″ value of CPO‐Alg gel matrix formed by different surface charge of CPO NPs in the time‐sweep test.

The CPO‐Alg hydrogels were fabricated through a two‐step process (**Figure** **1**A). First, 1.5% sodium alginate and dilute CPO solution were mixed evenly to form a gel matrix. Subsequently, the matrix was transferred to some volume of high concentrated CPO solutions several times, and finally obtained CPO‐Alg hydrogels with intended CPO loadings and mechanical properties (Figure S7, Supporting Information). The fabricated hydrogels have been reported as nanoparticle network hydrogels (NNH) by Campea et.al,^[^ ^15^ ^]^ in which CPO NPs act as an essential structural component to construct the gel network. As shown from Cryo‐Scanning Electron Microscopy (Cryo‐SEM) in Figure 1B, the matrix possessed an interconnected 3D network structure with hierarchical pore patterns, which allows for the efficient absorption of wound secretions. Images at higher magnification further revealed the rough texture of the surface on the gel cross‐section, with abundant CPO NPs (Figure 1B). Besides, Fourier‐Transform Infrared Spectroscopy (FT‐IR) of the hydrogel revealed the distinctive peaks of sodium alginate (1594 and 1406 cm^−1^)^[^ ^16^ ^]^ and CPO NPs (831, 881, and 1115 cm^−1^),^[^ ^7a^ ^]^ which further substantiated the presence of NPs within the gel (Figure 1C). The spectrum of solid sodium alginate showed the absorption bands of carboxyl functional groups. Intense bands observed at 1594 and 1406 cm^−1^ were attributed to asymmetric and symmetrical stretching vibrations in the carboxylate ions. The bands between 1100 and 950 cm^−1^ were attributed to the C─O stretching vibration as well as the deformation of the C─C─H and C─O─H bonds. Comparatively, CPO NPs exhibited absorption band characteristics at around 831, 881, and 1115 cm^−1^, which can be due to peroxyl bond (O─O). Additionally, the confocal laser scanning microscope (CLSM) images also confirmed the successful preparation of the hydrogels, as evidenced by the presence of sulforhodamine B‐labelled CPO NPs (Figure S8, Supporting Information).

Ionic interactions and electrostatic interactions are the two major mechanisms responsible for the formation of CPO‐Alg gel matrix.^[^ ^17^ ^]^ Specifically, positively charged NPs were first attracted to the negatively charged alginate macromolecules through physical interactions, primarily electrostatic and hydrogen bonding. Subsequently, carboxyl groups on the alginate chains would engage in further ionic cross‐linking with calcium ions exposed on the surface of NPs. In a simplified model alternative, each CPO nanoparticle can be regarded as up‐scaled calcium aggregates that undergo gelation with alginate. To validate the hypothesis, the CPO NPs were subjected to surface modifications, with the intention of altering the surface charges of NPs as well as masking the surface‐exposed calcium ions. As shown in Figure 1D–F and Figure S9A‐B (Supporting Information), in contrast to the positively charged NPs formed the gel matrix, the negatively charged NPs failed to make complete gelation. The strain‐sweep rheological test disclosed that hydrogels fabricated with positively charged NPs exhibited the highest Storage modulus (G′) value (Figure 1F; Figure S10, Supporting Information). Conversely, hydrogels formulated with NPs bearing surface charges of −15.5 and −21.4 mV displayed inferior G′ values to their shear loss modulus (G″) values, indicating that the fluid state persists and hydrogel formation is impeded (Figure 1D–F). Additionally, time‐sweep rheological test showed that the time point for gelification begins was ≈10,30, and 90 min when using NPs charged with +25.8, −15.5, and −21.4 mV, respectively (Figure 1G–I). This further emphasized the importance of electrostatic and ionic interactions during the gelation process.

**Figure 2:**
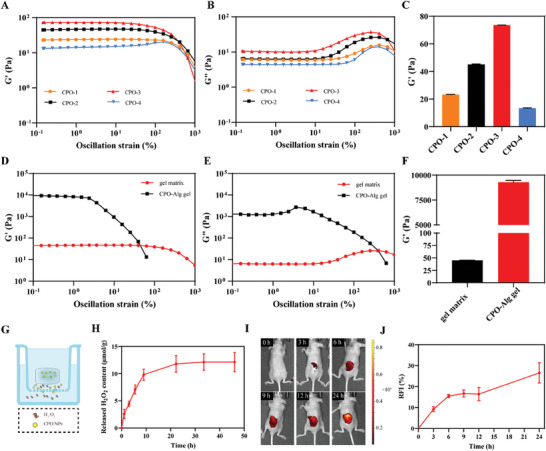
Optimization and characterizations of CPO‐Alg gel. A) G′ value of CPO‐Alg gel matrix formed by different particle sizes of CPO NPs in the strain‐sweep test. B) G″ value of CPO‐Alg gel matrix formed by different particle sizes of CPO NPs in the strain‐sweep test. C) G′ value of CPO‐Alg gel matrix formed by different particle sizes of CPO NPs at strain of 1%. The hydrodynamic size of CPO‐1, CPO‐2, CPO‐3, and CPO‐4 is 45, 68, 93, and 169 nm, respectively. D,E) G′ and G″ value of CPO‐Alg gel matrix and CPO‐Alg gel in the strain‐sweep test. F) G′ value of CPO‐Alg gel matrix and CPO‐Alg gel at strain of 1%. G) Illustration of H_2_O_2_ release pattern of gel. H) Release curve of H_2_O_2_ at different time points. I) Intravital fluorescence imaging in vivo at different time points after covering fluorescently labeled CPO‐Alg gel on the skin. J) Quantification of CPO NPs release in vivo for CPO‐Alg gel (n = 3 independent samples).

By incorporating NPs directly into the network, the mechanical properties of CPO‐Alg hydrogels can be manipulated more effectively, leveraging the available variety of NPs in terms of size, shape, and functional modification (Figure S11, Supporting Information). As depicted in **Figure** **2**A–C, rheological testing revealed that NPs of varying particle sizes significantly influenced the shear storage modulus, with those sized 93 nm demonstrating the highest mechanical strength. Besides, to achieve effective gelation, it is essential that the quantity of NPs formulated have to exceed a particular content level. As depicted in Figures S12A–C and S13A (Supporting Information), varying volume ratios of CPO NPs and Alg solution were employed to further optimize the physical properties of hydrogels. the hydrogel formed at a 1:1 ratio exhibited the highest G′ value. Consequently, the ratio was fixed for subsequent experiments.

The as‐prepared gel matrix was further incubated sequentially with concentrated solutions of CPO NPs, resulting in the formation of supersaturated carboxyl‐Ca^2+^ coordination complexes that act as the additional cross‐links to strengthen the gel matrix.^[^ ^18^ ^]^ The mechanical performance of the resultant CPO‐Alg hydrogels were markedly superior to the one before, with the G′ values increasing by 2–3 orders of magnitude (Figure 2D–F). Moreover, the incubation process also expanded the loading capacity of the hydrogel, with the CPO NPs acting not only to shape the structure but also as antibacterial agents. The resulted CPO‐Alg hydrogels facilitate the coexistence of CPO NPs with two distinct coordination states in the gel structure: one state (CPO^**^) that form strong coordination with alginate macromolecules, and the other state (CPO^*^) who being adsorbed on the polymer chains with weak interactions or as free form inside the gel matrix. The hydrogel formation process was further studied by molecular dynamics simulation. The simulation demonstrated the existence of two kinds of CPO NPs in the hydrogel with two distinct coordination states (Figure S14A,B, Supporting Information). Radial distribution function (RDF) calculation indicated that CPO^**^ had stronger interactions with the oxygen atoms of carboxyl groups than CPO^*^, especially within the long range of the distance (r >6 Å). Comparatively, the peak for CPO^*^ is lower, signifying weaker interactions with the oxygen atoms.

While CPO^*^ could be diffused out of the hydrogels, CPO^**^ under strong chemical bonding unable to detach from the gel, but slowly break down by water erosion that producing small amount of H_2_O_2_ with a sustained and controlled release manner (Scheme 1). This kind of release pattern could largely alleviate the negative concerns of local H_2_O_2_ accumulation with unexpected rapid release. To assess H_2_O_2_ release, gels were placed in the upper chamber of a 12‐well transwell plate, and the H_2_O_2_ content was measured in the lower chamber at different time points at room temperature, as depicted in Figure 2G. The concentration of H_2_O_2_ surged to 80% of the total released amount within 10 h and subsequently entered into plateau period in 48 h (Figure 2H). Besides, the NTA analysis also recorded the presence of CPO NPs in the lower chamber in 5 h (Figure S15, Supporting Information). In addition, hydrogels formulated by fluorescent sulforhodamine B labelled CPO NPs were topically administered to mouse skin to further verify the release of NPs from the gel. The fluorescence intensity within the treated area progressively enhanced over time, with a cumulative release of 27% achieved within 24 h (Figure 2I–J). These collectively indicated the release of CPO NPs from CPO‐Alg hydrogels.

### 2.2 Antibacterial Effect of CPO‐Alg Gel In Vitro

**Figure 3:**
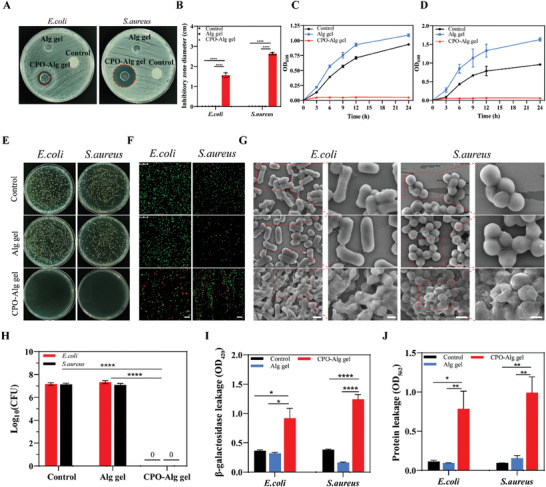
The antibacterial ability of CPO‐Alg gel in vitro. A, B) Photographs (A) of inhibition zones and quantitative analysis (B) of inhibitory zone diameter with different treatments. C,D) Effects of different treatments on the growth curves of *E. coli* (C) and *S. aureus* (D). E) Photographs of bacterial colonies of *S. aureus* and *E. coli* on agar plates after different treatments. F) Fluorescence images of live/dead bacterial staining in different treatment groups. Scale bar: 15 µm. G) SEM images of *E. coli* and *S. aureus* after different treatments. Scale bar, 1 µm (left) and 0.5 µm (enlarged). H) Antibacterial efficiency of Alg gel and CPO‐Alg gel against *E. coli* and *S. aureus* was determined by CFU assay. I,J) β‐galactosidase (I) and bacterial protein (J) leakage levels after different treatments. (n = 3 independent samples). *P* value: *p < 0.05, **p < 0.01, ***p < 0.001, ****p < 0.0001.

Following with the favored cascade release behavior of H_2_O_2_, antimicrobial performance of CPO‐Alg gel was assessed using *Escherichia coli* (*E. coli*) and *Staphylococcus aureus* (*S. aureus*) as model strains. Given the established minimum inhibitory concentrations (MIC) of CPO NPs against *E. coli* and *S. aureus*, which were 2  and 0.5 mM respectively (Figure S16 and Table S1, Supporting Information), inhibition zone assays were applied to determine the bacteria susceptibility to hydrogel sheets. Results depicted in **Figure** **3**A,B revealed a distinct “zone of inhibition” around CPO‐Alg gel for both *E. coli* (1.6 ± 0.1 cm) and *S. aureus* (2.6 ± 0.1 cm), whereas no such zone observed around control and Alg gel groups, demonstrating the remarkable antimicrobial performance of CPO‐Alg gel. Furthermore, while the growth curve analysis indicated a significant increase in OD_600_ values over time in control and Alg gel groups, the OD_600_ values co‐incubated with CPO‐Alg gel remained farther down for both *E. coli* and *S. aureus*, suggesting an effective growth inhibition (Figure 3C,D). The Alg gel group showed a faster increase in OD_600_ values than the control group, probably due to the 3D porous structure of Alg gel facilitating oxygen, nutrient, and metabolite transport. Thus, bacteria can grow faster within the Alg gel, while CPO‐Alg gel can inhibit bacterial growth due to the continuous release of H_2_O_2_. Subsequent plate counting assay confirmed the bactericidal efficacy of CPO‐Alg gels, with no visible bacterial colonies appeared on agar plates, indicating a sterilization rate exceeding 99.999% (Figure 3E,H). Bacterial viability was further validated through bacterial live/dead staining, where the CPO‐Alg group exhibited significantly higher red fluorescence compared to the control group and Alg gel group, indicating effective eradication of both bacterial strains (Figure 3F). The microstructural alterations of bacteria were also verified via SEM. As shown in Figure 3G, *E. coli* and *S. aureus* treated with PBS or Alg gel displayed typical rod‐shaped and spherical morphologies with intact and smooth surfaces. In contrast, co‐incubation with CPO‐Alg gel resulted in wrinkling and rupture of the bacterial surfaces, indicative of severe cell membrane damage and leakage of cellular contents, including enzymes, proteins, and nucleic acids.

To further assess the impact of CPO‐Alg gel on changes in bacterial cell membrane permeability, an o‐nitrophenyl‐β‐D‐galactopyranoside (ONPG) assay was performed to detect β‐galactosidase of supernatant after different treatments.^[^ ^19^ ^]^ Disruption of the bacterial cell membrane led to the release of β‐galactosidase, which then hydrolyzed ONPG into o‐nitrophenol (ONP) that could be identified at 420 nm. The OD_420_ of the CPO‐Alg gel group was significantly higher than that of the control and Alg gel groups (Figure 3I), indicating altered bacterial cell membrane permeability and leakage of β‐galactosidase. Furthermore, protein leakage in the supernatant after 12 h of gel incubation further matched the ONPG assay results, with the CPO‐Alg gel group exhibiting significantly higher protein content than the other two groups (Figure 3J). These findings confirmed that the CPO‐Alg gel exerts its prominent antimicrobial functionality by impairing bacterial cell walls and membranes, which in turn leads to the leakage of cytoplasm.

### 2.3 Anti‐Biofilm Activities of CPO‐Alg Gel In Vitro

**Figure 4:**
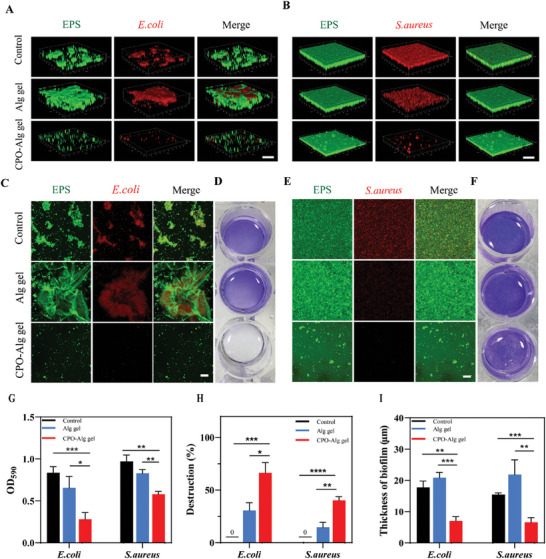
Evaluation of anti‐biofilm activities by CPO‐Alg gel. A,B) 3D reconstruction of confocal images of the *E. coli* (A) biofilm and *S. aureus* (B) biofilm in different treatment groups. Green: EPS, Red: bacteria. Scale bar, 50 µm. C,E) Confocal images of the *E. coli* (C) biofilm and *S. aureus* (E) biofilm in different treatment groups. Green: EPS, Red: bacteria. Scale bar, 25 µm. D, F) Photographs of the crystal violet staining of *E. coli* (D) and *S. aureus* (F) biofilm in different treatment groups. G,H,I) Corresponding OD 590 value of crystal violet (G), destruction rate (H), and thickness (I) of biofilm of each group (n = 3 independent samples). *P* value: *p < 0.05, **p < 0.01, ***p < 0.001, ****p < 0.0001.

Biofilm represents a kind of survival strategy for bacteria during their developmental stages to adapt environmental shifts, characterized by their attachment to both living or non‐living surfaces.^[^ ^20^ ^]^ Biofilms consist of bacteria embedded in extracellular matrix, which composed of proteins, polysaccharides, nucleic acids, and other organic compounds. Serving as a self‐defense mechanism for bacteria, the formation of biofilms often impedes antibacterial effect and leads to bacterial resistance.^[^ ^21^ ^]^ Motivated by the robust capability of CPO‐Alg gel to eradicate planktonic bacteria, we further examined their biofilm elimination potential using FITC‐ConA/PI dual‐staining. FITC‐ConA could attach to polysaccharides in the extracellular matrix, while propidium iodide (PI) prone to bind the cellular DNA of bacterial. As depicted in **Figure** **4**A,B, both the control group and Alg gel group exhibited extensive and unbroken biofilms, with bacteria embedded within a densely structured matrix. In contrast, the CPO‐Alg gel group showed scattered bacteria and a reduced amount of extracellular polysaccharide matrix (Figure 4C,E). The thickness of biofilms was further evaluated by 3D confocal microscopy. As shown in Figure 4I, the thickness of *E. coli* and *S. aureus* biofilms in the CPO‐Alg gel group was 7.0 ± 1.4 and 6.6 ± 1.5 µm, respectively. These thicknesses were notably thinner compared to those in the control group (*E. coli*: 17.7 ± 2.1 µm, *S. aureus*: 15.5 ± 0.5 µm) and the Alg gel group (*E. coli*: 20.8 ± 1.8 µm, *S. aureus*: 21.8 ± 4.8 µm). Additionally, the quantitative analysis of biofilm by crystal violet staining also validated the elimination of biofilm (Figure 4D,F). The removal rates of *E. coli* and *S. aureus* biofilms by CPO‐Alg gel were 66% and 40%, respectively. It's worth noting that Alg gel also exhibited a certain anti‐biofilm capacity (*E. coli* biofilm: 22%, *S. aureus* biofilm: 15%), which possibly due to the dragging effect during gel removal resulting in biofilm detachment (Figure 4G,H). These results collectively indicated the excellent biofilm removal performance of CPO‐Alg gel.

### 2.4 Antibacterial Mechanism of CPO NPs

**Figure 5:**
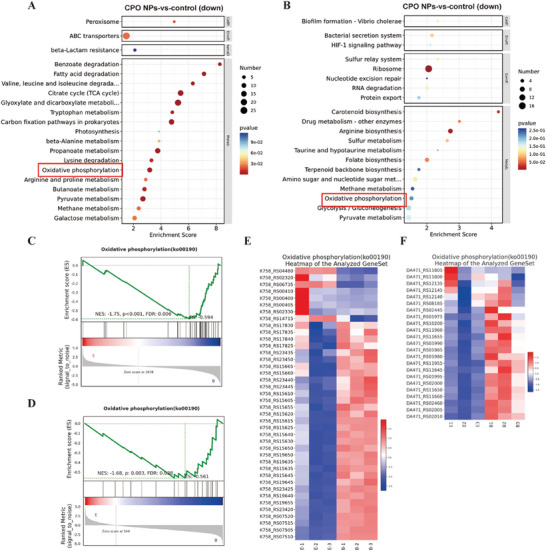
Antibacterial mechanism mediated by CPO NPs. A) KEGG enrichment analysis of DEGs in *E. coli* after treatment with PBS and CPO NPs. B) KEGG enrichment analysis of DEGs in *S. aureus* after treatment with PBS and CPO NPs. C) GSEA analysis of oxidative phosphorylation pathway in *E. coli* after treatment with PBS and CPO NPs. D) GSEA analysis of oxidative phosphorylation pathway in *S. aureus* after treatment with PBS and CPO NPs. E) Heatmaps of DEGs in oxidative phosphorylation pathways in *E. coli* treated with PBS and CPO NPs. F) Heatmaps of DEGs in oxidative phosphorylation pathways in *S. aureus* treated with PBS and CPO NPs.

RNA sequencing (RNA‐seq) transcriptomics was performed to in‐depth investigate the mechanism behind the excellent antibacterial ability of CPO NPs. Gene expression profiles of *E. coli* and *S. aureus* treated with PBS and CPO NPs were analyzed. The differentially expressed genes (DEGs) between the two groups were showed through the volcano plot. Compared with the control group, 422 DEGs were upregulated and 424 DEGs were downregulated in the genome of *E. coli* treated with CPO NPs (Figure S17, Supporting Information). In addition, a total of 693 DEGs including 418 upregulated DEGs and 275 downregulated DEGs were observed in the genome of *S. aureus* treated with CPO NPs (Figure S17A,B, Supporting Information). To further determine which of these DEGs were involved in the internal physiological activities of bacteria, Gene ontology (GO) analysis and Kyoto Encyclopedia and Genes and Genomes (KEGG) enrichment analysis were performed. GO analysis demonstrated that these DEGs in the CPO NPs group were associated with translation and amino acid metabolism (glycine, serine, threonine, valine, leucine, isoleucine, tryptophan, and lysine). In addition, at the cellular component part, these DEGs were mostly enriched in cell components associated with cell wall, cell membrane, and ribosome, indicating CPO NPs could impair bacterial cell walls and membranes that consistent with previous results (Figures S18 and S19, Supporting Information). Additionally, the KEGG analysis also validated that the DEGs were mainly enriched in amino acids synthesis and metabolism‐related pathways (Figure **5**A,B). Furthermore, we found that genes involved in oxidative phosphorylation pathways were downregulated in both *E. coli* and *S. aureus* genomes after CPO NPs treatment (Figure 5A,B). Thus, Gene Set Enrichment Analysis (GSEA) was conducted to verify whether the oxidative phosphorylation pathway downregulated in both *E. coli* and *S. aureus* treated with CPO NPs. As shown in Figure 5C,D, the overall level of oxidative phosphorylation pathway was significantly downregulated in CPO NPs treated bacteria.

In addition, hierarchical clustering heatmap of oxidative phosphorylation pathway related DEGs demonstrated the genes encoding oxidative phosphorylation process related enzymes, such as *EcolC_4261* (encoding ATP synthase)*, c0541* (encoding cytochrome o ubiquinol oxidase)*, SF2356* (encoding NADH‐quinone oxidoreductase) and *atpD* (encoding ATP synthase)*, qoxC* (encoding cytochrome aa3 quinol oxidase)*, qoxB* (encoding cytochrome aa3 quinol oxidase), were significantly downregulated in *E. coli* and *S. aureus* genomes respectively compared with the control group (Figure 5E,F). The down‐regulation of these related genes further confirmed that CPO NPs could kill both Gram‐positive and ‐negative bacteria by interfering with the oxidative phosphorylation pathway. Collectively, it is speculated that CPO NPs are favorable for eliminating both Gram‐positive and negative bacteria through disrupting the process of oxidative phosphorylation and amino acids synthesis and metabolism.

### 2.5 Antibacterial and Wound Healing Evaluation In Vivo

**Figure 6:**
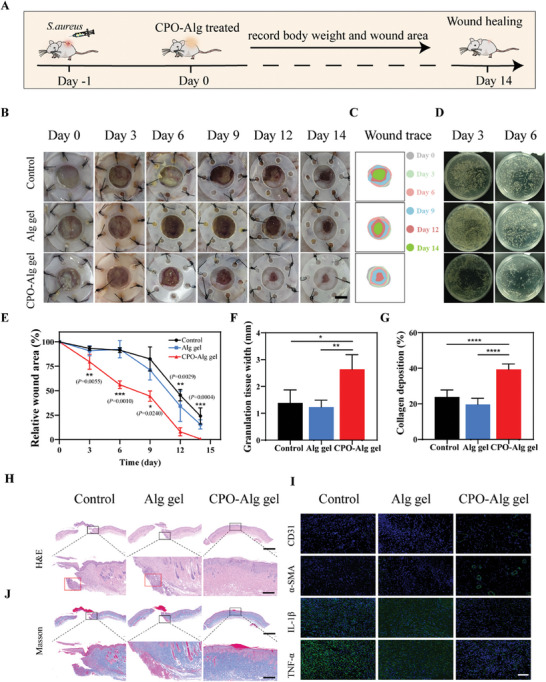
Performance of CPO‐Alg gel for antibacterial capability in mice full‐thickness skin excisional wound model infected with *S. aureus* infection. A) Schematic illustration of the establishment and treatment of skin excisional wounds by CPO‐Alg gel. B) Representative photographs of wound areas at different time points after varied treatments. Scale bar, 5 mm. C) Schematic diagram of wound shrinkage at different time points after varied treatments. D) Bacterial colonies of *S. aureus* on TSB agar plates in the wounds on day 3 and 6 post‐treatments (n = 5 biologically independent samples). E) Quantitative analysis of the relative wound areas at different times (n = 5 biologically independent samples). F) Quantitative analysis of granulation tissue width on day 14. G) Quantitative analysis of percentages of collagen on day 14. H) Representative H&E staining images of wounds on day 14. Scale bar, 2000 µm (up) and 400 µm (enlarged). (n = 3 biologically independent samples). I) Immunofluorescence images of CD31, α‐SMA, IL‐1β, and TNF‐α in different groups. Scale bar, 80 µm. J) Representative Masson Trichrome staining images of wounds on day 14. Scale bar, 2000 µm (up) and 400 µm (enlarged). (n = 3 biologically independent samples). *P* value: *p < 0.05, **p < 0.01, ***p < 0.001, ****p < 0.0001.

Encouraged by the excellent in vitro antimicrobial efficacy of CPO‐Alg gel, a full‐thickness dermal injury mouse model was established to evaluate its potential of promoting wound healing in vivo. *S. aureus* was inoculated into the wound regions in advance to instigate bacterial infections (**Figure** **6**A). Then, the mice were segregated into three groups, with the wound area receiving treatment with PBS, Alg gels, and CPO‐Alg gels, respectively. Alterations in body weight and the progress of wound healing for each group were monitored. As shown in Figure 6B, on day 0, the wounds exhibited yellow purulent exudate with a foul odor, indicating the effective formation of infected wounds. During the observation period, the wound area in all groups were progressively reduced over time, and the body weight got steadily increased (Figure 6C; Figure S20, Supporting Information). While the healing rate in the Alg gel group was similar to that in the control group, the CPO‐Alg gel group showed a significantly accelerated wound healing rate (Figure 6B,E), with a marked reduction in wound area observed on day 6. Additionally, the wounds in the control and Alg gel groups appeared dark yellow, with noticeable yellow fluid seeping out, while the CPO‐Alg gel group exhibited no apparent signs of infection, attributing to the robust antibacterial capability of CPO‐Alg gels, which was further confirmed by subsequent wound secretion spread‐plate tests. As shown in Figure 6D, the bacterial counts in each group were decreased over time. Notably, on day 6, the bacterial counts in the CPO‐Alg gel group were significantly lower compared to the other two groups. The wounds in the CPO‐Alg gel group were nearly completely healed by day 14, whereas mice in the control and Alg gel groups still displayed partially wounds. Throughout the experiment, the wound healing rate in the CPO‐Alg gel group was significantly superior to that in the control and Alg gel groups. This underscores the outstanding bactericidal effect of CPO‐Alg gels, which evidently boost the healing progression of infected wounds.

On day 14, samples of the wound areas were harvested for histopathological evaluation to assess tissue regeneration and inflammatory response within the infected lesions. Hematoxylin and eosin (H&E) staining revealed multiple inflammatory cells in skin tissue of the control group, while the number of inflammatory cells (indicated in red dash box in Figure 6H; Figure S21, Supporting Information) reduced with CPO‐Alg gel treatment (Figure 6H). Moreover, the CPO‐Alg gel group displayed the thickest granulation tissue, highest skin maturity, and exhibited histological structures similar to the surrounding normal skin tissues, including the existence of sebaceous glands, hair follicles, and sweat glands (Figure 6F,H). Masson staining demonstrated a significant increase of collagen deposition after CPO‐Alg gel treatment, with a collagen volume fraction reaching 40%, while the control and Alg gel groups exhibited collagen volume fractions of only 24% and 20%, respectively (Figure 6G,J).

To further evaluate the degree of inflammation at the wound site, an extensive analysis of pro‐inflammatory cytokine levels was conducted. Interleukin‐1β (IL‐1β) and tumor necrosis factor‐alpha (TNF‐α) were chosen as prototypical pro‐inflammatory cytokines. Immunofluorescence staining for IL‐1β and TNF‐α demonstrated significantly lower expression levels in wounds treated with CPO‐Alg gel compared to the other groups (Figure 6I). Besides, neoangiogenesis, which is essential for supplying adequate oxygen and nutrients to compromised tissues, also plays a pivotal role in the repair of wounds. Markers including CD31 and alpha‐smooth muscle actin (α‐SMA) were utilized to assess vascularization at the wound site. The expression of CD31 and α‐SMA in the CPO‐Alg gel treatment group was significantly higher than in the other two groups, and a clearer vascular lumen structure could be observed. The ability of promoting new blood vessels formation by CPO‐Alg gel could be due to the release of calcium ions from the gel. Calcium ions are involved in the process of angiogenesis and the formation of new blood vessels.^[^ ^22^ ^]^ Additionally, calcium ions are integral to multiple stages of wound healing, from hemostasis to tissue remodeling.^[^ ^23^ ^]^ Collectively, these results suggested the potent antibacterial activity of CPO‐Alg gel in mitigating wound infection, and its additional ability to foster collagen deposition and neoangiogenesis and thereby accelerating infected wounds healing.

Finally, in vivo biosafety of CPO‐Alg gels was systematically evaluated. Mice venous blood samples were collected after 14 days of the treatment. As depicted in Figure S22 (Supporting Information), the hematological markers, including white blood cell counts (WBC), red blood cell counts (RBC), and platelet counts (PLT), in the CPO‐Alg gel group were comparable to those in the control and Alg gel groups, indicating favorable biocompatibility of CPO‐Alg gel. Concurrently, mice serum was collected for the assessment of hepatic function markers (alanine aminotransferase (ALT), aspartate aminotransferase (AST)) and renal function markers (creatinine (CREA)). As illustrated in Figure S23 (Supporting Information), in comparison to the control group and Alg gel group, no significant alterations were observed in serum levels of ALT, AST, and CREA when treated with CPO‐Alg gel, suggesting the normalcy of liver and kidney functions following CPO‐Alg gel treatment. Moreover, mice were sacrificed and the major organs (heart, liver, spleen, lungs, and kidneys) were collected for comprehensive systemic toxicity evaluation. Histological analysis revealed no apparent damage or inflammatory lesions (Figure S24, Supporting Information). These cumulative findings described the desirable biocompatibility and safety profile of CPO‐Alg gel, indicating its potential as a safe and effective wound dressing for wound healing applications.

## 3 Conclusion

In summary, as a carrier for H_2_O_2_ sustained release, CPO NPs perfectly matches the biological requirement of H_2_O_2_ for wound healing. High concentrations of CPO NPs release a large amount of H_2_O_2_ to strongly eliminate bacteria, while low concentrations of CPO NPs exert regulatory effects in physiological environments, promoting the proliferation and growth of tissue cells. The electrostatic interaction between CPO NPs and the abundant carboxyl groups in sodium alginate cross‐links to form a new CPO‐Alg hydrogel, significantly enhancing biocompatibility and retention at the wound site. Importantly, such special structure of hydrogel achieves secondary sustained release of CPO NPs, allowing easy regulation of CPO NPs concentration for on‐demand release of H_2_O_2_ for promoting wound healing. Both in vitro and in vivo studies revealed the mechanism of forming CPO‐Alg hydrogel as well as its antibacterial activity and promotion of wounds healing. Therefore, the developed CPO‐Alg hydrogel holds promise as an antibacterial strategy, which may potentially reshape traditional antibiotic therapies.

## 4 Experimental Section

### Materials

Calcium chloride hexahydrate (CaCl_2_·6H_2_O), sodium alginate, 30% hydrogen peroxide (H_2_O_2_) solution, methanol (CH_3_OH), ammonia solution, calcium carbonate (CaCO_3_), and d‐glucono‐d‐lactone (GDL) were purchased from Macklin. Arginine and lysine were obtained from TCI. 4% polyformaldehyde (PFA), phosphate buffered solution (PBS), crystal violet staining solution (1%), sodium citrate, and sulforhodamine B sodium salt were purchased from Adamas. Cerium sulfate standard solution (0.1000 mol L^−1^) was purchased from Aladdin. BCA protein assay kit and ortho‐nitrophenyl‐β‐galactoside (ONPG) were purchased from Beyotime. SYTO 9 and Propidium iodide (PI) were obtained from Thermo Fisher. Fluorescein isothiocyanate conjugated concanavalin A (FITC‐ConA) was bought from Sigma‐Aldrich. Luria‐Bertani (LB) medium, Tryptic Soy Broth (TSB) medium, LB agar, and TSB agar were bought from Hopebio.

### Bacteria and Animals

*Staphylococcus aureus* (ATCC 6538) and *Escherichia coli* (ATCC 8739) were purchased from China General Microbiological Culture Collection Center. Animal experiments were approved by the animal care and use committee at Shanghai Jiao Tong University School of Medicine and performed according to the National Institutes of Health Guidelines (A2024140).

### Characterization

Scanning electron microscope (SEM) images were obtained from JEOL JSM‐7800F and ZEISS Gemini 300. TEM. The hydrodynamic radius and zeta‐potential were measured by Brookhaven omni. X‐ray diffraction (XRD) patterns were collected by Rigaku D/MAX‐2250 V. Fourier transform infrared (FT‐IR) spectra were obtained using Thermo Fisher Nicolet 6700. Confocal laser scanning microscopy (CLSM) images were conducted on Leica TCS SP8 STED 3X. Animal imaging of mice in vivo was detected by PerkinElmer IVIS Spectrum Imaging System.

### Rheological and Viscosity Test

The storage modulus (G′), loss modulus (G″), viscosities, and stress were measured using rotational rheometer (TA DHR20/10). G′ and G″ values were measured within different oscillation strain (0.1%−1000%) at constant temperature (25 °C) and constant frequency (10 rad s^−1^). Viscosity and stress were subsequently tested at different shear rates (0.01–100 s^−1^).

### Synthesis of CPO NPs

CPO NPs were prepared according to previous procedures with slightly modifications.^[7a]^ Typically, 1 mmol of hydrogen peroxide solution (10 mol L^−1^), 2 mL of ammonia solution (8% w/v), and 15 mg of lysine were added to a 10 mL of vigorously stirred absolute methanol. The reaction was initiated by slowly addition 0.5 mL of the calcium chloride hexahydrate (50 mg mL^−1^) dropwise and mixing. About 10 min later, the reaction was terminated, and the solution was ultracentrifuged with 20 000 g for 20 min and then dialyzed twice with deionized water. To obtain CPO products with different nanoparticle size, the amount of ammonia added was adjusted to 1, 1.5, and 2.5 mL. The products were finally dispersed in a dedicated formulation buffer and stored at −80 °C after detecting the containing concentration of hydrogen peroxide by the cerium sulfate method.^[7a]^

To get negatively charged CPO NPs, 1 mmol NPs were redispersed in 15 mL of DI water, followed by the addition of 13, 26, 39, 52, and 78 mg of sodium citrate solution (100 mg mL^−1^) into the solution, respectively. The mixture was fully mixed under magnetic stirring at 4 °C. Subsequently, the solution was centrifuged and washed twice with DI water. The final product was dispersed in 5 mL DI water.

### Fabrication of Alg Hydrogel and CPO‐Alg Hydrogel

For Alg hydrogel formation, 1.5% sodium alginate solution and 25 mM CaCO_3_ suspension were prepared before they were mixed together to form a cloudy solution. A fresh aqueous d‐glucono‐d‐lactone (GDL) solution was then added to the mixture and vortexed to initiate gelation. A CaCO_3_ to GDL molar ratio of 0.5 was maintained to achieve a neutral pH post‐cross‐linking.

For CPO‐Alg hydrogel fabrication, CPO working solution (25 mM) was rapidly mixed to 1.5% sodium alginate solution with the volume ratio of 2:1, 3:1, 1: 1, 1:2, 1:3, and 1:4. The mixture was ultrasonicated and left 4 h at room temperature for further cross‐linking to obtain the gel matrix. The gel matrix was immersed in the 20 mL CPO working solution (50 mM) three times (ensuring that the gel matrix was fully immersed each time) to obtain the final CPO‐Alg hydrogel. The final hydrogel was lyophilized for further use.

### Molecular Dynamics (MD) Simulation—Model Construction

The initial model was built using the 3D view module in Materials Studio Charge Assignment and Geometry Optimization: Positive charges were assigned to Ca_10_O_20_ (CPO) and negative charges to sodium alginate after removing Na atoms. Geometry was optimized using the Universal Force Field (UFF), with charges calculated by the QEq method (energy deviation 10^−4^). The Ewald method was used for summing van der Waals and electrostatic forces.

### Molecular Dynamics (MD) Simulation—Mixed Model Construction and Simulation (Gel Matrix Formation)

A mixed model with 4800 water molecules, 64 CPO (noted as CPO^**^), and 16 sodium alginate units were built using the Amorphous Cell module for dynamic simulation. The NVE ensemble at 298K, total simulation time of 5 ns, UFF, and a cut off radius of 9.5 Å were used.

### Molecular Dynamics (MD) Simulation—Second Mixed Model Construction and Simulation (CPO‐Alg Hydrogel Formation)

An additional 64 CPO (noted as CPO^*^) were added to the simulation results to construct a second mixed system. Dynamic simulations were performed under the same conditions as the first system.

### Release of CPO NPs and H_2_O_2_ In Vitro

A certain mass of lyophilized gel was weighed and placed in the upper chamber of 12‐well transwell. Then, 0.9 mL buffer solution was added to the upper chamber, and 2.1 mL buffer was added to the lower chamber of the transwell. At the present time point (1, 3, 5, 9, 22, 33, and 46 h), certain amount of liquid in the lower chamber was pipetted for H_2_O_2_ detection. Besides, the solution in the lower chamber after immersion of the gels for 5 h were then detected using Nanoparticle tracking analyzer (Particle Metrix Zetaview TWIN) to detect the release of CPO NPs from the gel.

The release amount of H_2_O_2_ was measured by the cerium sulfate method, as reported by previous protocol.^[7a]^ Briefly, Ce (IV) ions could be stoichiometrically reduced to colorless Ce (III) by H_2_O_2_ in acidic solution. While the concentration of Ce (IV) ions was linearly related to the absorbance at 350 nm within a certain range, and the consumed Ce (IV) ions could be measured by using multifunctional microplate reader.

### Release of CPO NPs In Vivo

CPO NPs were first labelled with sulforhodamine B (Ex/Em: 565/586 nm). Briefly, sulforhodamine B (1 mg mL^−1^) and CPO NPs solution (25 mM) were mixed and stirred for 2 h at room temperature, and the resultant products were collected by centrifugation and washing with deionized water. Sulforhodamine B‐labelled CPO‐Alg hydrogel was then prepared as described above. To investigate the in vivo release of CPO NPs, fluorescently labeled CPO‐Alg hydrogel was affixed to the back of the mice. The gel was removed during mouse imaging. Mice were imaged using an in vivo spectrum imaging system at different time points (0, 3, 6, 9, 12, and 24 h).

### Bacterial culture

The cryopreserved strains (*E. coli* and *S. aureus*) were streaked into plate medium to obtain single colonies (37 °C, 24 h). Then single colonies were inoculated into liquid medium, incubated in an incubator shaker overnight (37 °C, 200 rpm). The bacterial suspension was centrifuged for 5 min at 4000 rpm and washed three times with sterile PBS, then diluted to the experimental concentration for further use.

### Inhibition Zone Test

The bacterial suspension (1 × 10^8^ CFU mL^−1^) was evenly spread on the agar plate with a cotton swab and the hydrogel sheet was gently placed on the surface of the agar plate. The inhibition zone diameter around the samples was measured after the plates were incubated overnight at 37 °C.

### The Minimum Inhibitory Concentration (MIC) Test of CPO

The MIC values against *E. coli* and *S. aureus* were determined by the protocol of the Clinical and Laboratory Standards Institute (CLSI) broth method. Briefly, *E. coli* and *S. aureus* (10^6^ CFU mL^−1^) were inoculated in LB and TSB respectively. CPO NPs were dispersed in LB or TSB broth and tested at a final concentration of 31.25–4000 µ. A mixture of CPO (50 µL) and bacteria (50 µL) was added to the 96‐well plate and incubated at 37 °C for 12 h. The positive control contained bacteria without CPO NPs, while the negative control contained only broth without CPO NPs and bacteria. MIC was determined as the lowest dilution of a complex that produced no visible turbidity. All experiments were performed in parallel for three times.

### Growth Curve Inhibition Assay

To measure growth curve inhibition assay, 1 g hydrogel was placed in a 5 mL centrifuge tube, and then 1 mL of bacterial suspension (1 × 10^5^ CFU mL^−1^) was placed above the hydrogel, which was finally incubated in a shaker (37 °C, 200 rpm). After incubation for 0, 3, 6, 9, 12, and 24 h, the bacterial suspension above the gel was taken to 96‐well plate, and OD_600_ was measured to determine the growth of bacteria.

### The Plate Counting Method

The bacterial suspension (1 mL, 1 × 10^7^ CFU mL^−1^) was incubated with 1 g CPO‐Alg hydrogel or Alg hydrogel for 12 h. Then treated bacterial suspension was diluted appropriately. Afterward, 50 µL suspension was pipetted out and evenly coated on the solid medium, and finally incubated for 18 h at 37 °C before performing the colony counting.

### Bacterial Live/Dead Staining Assay and Morphology Observation

A suspension of *S. aureus* with OD_600_ = 0.5 and *E. coli* with OD_600_ = 0.3 was diluted 10 times with PBS and incubated with the gel for a certain time. After three washes with 0.85% NaCl, the bacteria were resuspended in 200 µL NaCl. Subsequently, SYTO 9 (5 µm) and PI (1 µg mL^−1^) were added to the suspension, which was stained for 15 min in the dark. Finally, the stained bacteria were observed under CLSM.

The destructive effect of CPO‐Alg gel on the bacterial cell wall was observed through SEM. Briefly, following the incubation of bacteria and gels, the bacteria were fixed with 2.5% glutaraldehyde for 4 h at 4 °C. Subsequently, the bacterial samples were subjected to gradient dehydration and dried on a silicon slice. Finally, the samples were observed under SEM.

### ONPG and Protein Leakage Test

The release of bacterial extracellular β‐galactosidase was measured using ONPG as a chromogenic substrate. Briefly, following the incubation of bacteria and gels, the treated bacterial suspensions were centrifuged (3000 g, 5 min) and the supernatant were collected. Subsequently, the supernatant was reacted with ONPG (1 mM) solution at 25 °C for 2 h, and the change in absorbance at λ_420_ was monitored with a multifunctional enzyme marker. In addition, the protein quantification of supernatants was detected by BCA protein assay kit to monitor protein leakage.

### Biofilm formation and anti‐biofilm activity of CPO‐Alg gel

To evaluate the ability of destructing mature biofilms by CPO‐Alg gel, 1 mL of bacteria suspension (10^6^ CFU mL^−1^) was added into a 24‐well plate prepped with square microscope coverslips and incubated at 37 °C for 48 h to obtain mature biofilms. To promote biofilm formation, the liquid medium was additionally supplemented with 1% glucose. Fresh liquid medium was replaced every 24 h. The mature biofilm was washed with PBS softly, and then received different treatments. Afterward, the plate was washed three times with PBS, incubated with anhydrous methanol for another 10 min, and finally stained with 500 µL 0.1% crystal violet for 10 min. The stained biofilms were washed three times with PBS. The absorbance at 590 nm was obtained using a multifunctional microplate reader when the stained biofilms were dissolved in 500 µL of 95% ethanol. Meanwhile, biofilms with different treatments were stained with FITC‐ConA and PI for 20 min at 4 °C, and then imaged by CLSM to obtain 3D structures of biofilms and the thickness of biofilm.

### Transcriptome Sequencing and Data Analysis

The bacteria after different treatments were collected and stored at −80 °C. Total bacterial RNA was extracted using the RNA Isolation Kit following the manufacturer's protocol. Gene profiles were analyzed by Oebiotech Co., Ltd. (Shanghai, China). The gene transcript data analysis, including the heatmaps, volcano plots for differently expressed genes (|fold change| ≥2.0; FDR < 0.05), and Kyoto Encyclopedia of Gene and Genomes (KEGG) analysis was performed using the Oebiotech cloud platform (https://cloud.oebiotech.cn/task/).

### Treatment for Infected Cutaneous Wound

Six‐week‐old Kunming mice were given general anaesthesia with isoflurane (RWD) to establish a bacterial infection model of skin wounds. While rodent wound healing was primarily driven by contraction, human wound healing involves re‐epithelialization and the formation of granulation tissue as the main mechanisms. Therefore, the murine model employed silicone splints to immobilize the wound periphery, ensuring a maximally accurate emulation of the human wound healing process. Briefly, after shaving the upper back region of the mice, they were sterilized with 75% ethanol, and the round whole skin with a diameter of 1.0 cm was excised. Then a silicone ring was sewn around the wound to splint the wound. 50 µL of *S. aureus* (10^8^ CFU mL^−1^) was then coated to the wound area on day −1. After 24 h of the infection, mice were randomly divided into three groups (noted as control group, Alg gel group, CPO‐Alg group), and treated with PBS, Alg gel, and CPO‐Alg gel, respectively. Gels were replaced every three days at the first week, and the wound area was photographed and measured. Bacteria from the wound were collected using sterile swabs and plate counting assay were performed to assess the bacterial burden. On day 14, the wounds for all groups were collected and stored in 4% PFA for H&E staining, Masson staining, and immunohistochemical staining.

### In Vivo Biocompatibility Assay

To test the biocompatibility in vivo, the Kunming mice (6 weeks, female) were treated with CPO‐Alg gel on the wound area. After 14 days treatment, fresh blood from mice was harvested under anesthetization for hematology test and blood biochemical test. A control group with PBS treatment was set. After euthanasia, the main organs (heart, liver, spleen, lung, and kidneys) of the mice were also obtained for H&E staining.

### Schematic Illustration Creation

Illustrations in Figure 1A (Agreement number: QS270TP5DI) were created with BioRender.com.

### Statistical Analysis

The GraphPad Prism software (version 8.2.1) and ImageJ software (version 2.1.0) were used for the statistical analysis. All data were obtained from at least three independent experiments with at least three parallel samples per condition in each experiment, which were expressed as mean ± standard deviations (SD). Multiple comparisons were performed using one‐way two‐sided analysis of variance (ANOVA) with Tukey's multiple comparison test. A probability value of *p* < 0.05 was considered statistically significant (*p < 0.05, **p < 0.01, ***p < 0.001, ****p < 0.0001).

## Conflict of Interest

The authors declare no conflict of interest

## Supporting information

Supporting Information

## Data Availability

The data that support the findings of this study are available from the corresponding author upon reasonable request.
