# Supplementary material for: Calcium Peroxide‐Based Hydrogels Enable Biphasic Release of Hydrogen Peroxide for Infected Wound Healing
Source: Adv Sci (Weinh). 2024 Sep 3;11(40):2404813. doi: 10.1002/advs.202404813 (PMC11516063; doi:10.1002/advs.202404813)
Supplement: Supplementary file 1 — Supporting Information [file ADVS-11-2404813-s001.docx]

Supporting Information

**Calcium Peroxide-based Hydrogels Enable Biphasic Release of Hydrogen Peroxide for Infected Wound Healing**

*Ying Huang^1^, Zi Fu^1^, Han Wang^1^, Zeyang Liu^1^, Mengqi Gao^2^, Yanran Luo^3^, Meng Zhang^1^*,* *Jing Wang^4^*, Dalong Ni^1^**


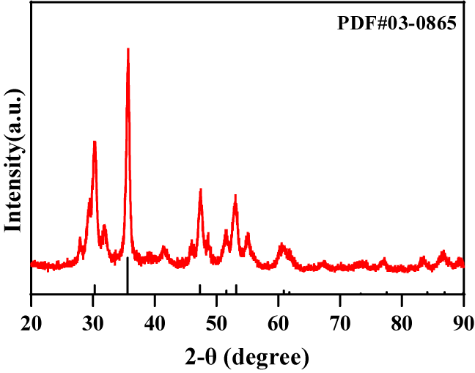


Figure S1: X-ray powder diffraction pattern of the CPO NPs.


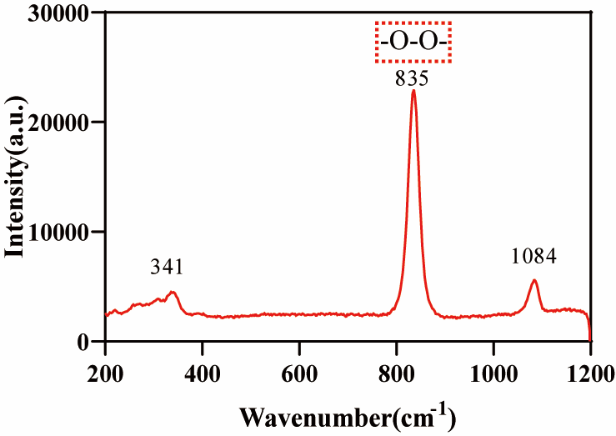


Figure S2: Raman spectra of the CPO NPs.


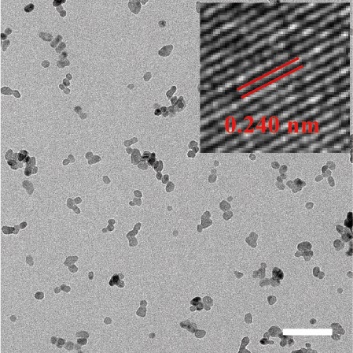


Figure S3: TEM images CPO NPs. Scale bar, 100 nm. Inset graph: HRTEM images of CPO NPs.


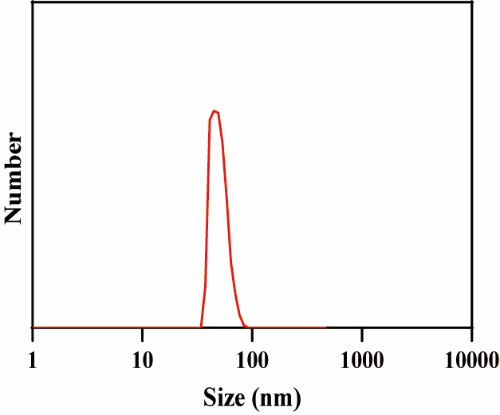


Figure S4: Hydrodynamic size of CPO NPs.


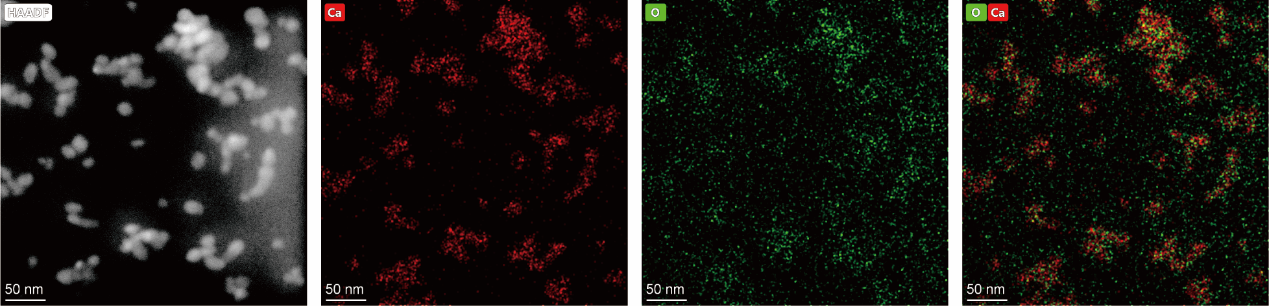


Figure S5: HADDF-STEM and elemental mapping images of CPO NPs. Scale bar: 50 nm.


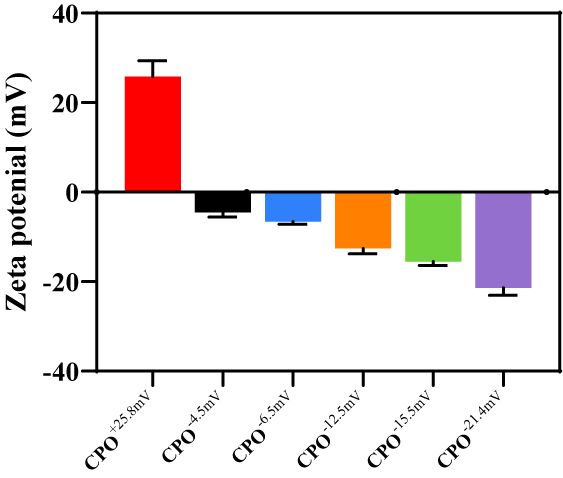


Figure S6: Zeta potential of modified CPO NPs after the surface functionalization with varying ratios of ligating agents (n=3, mean±SD).


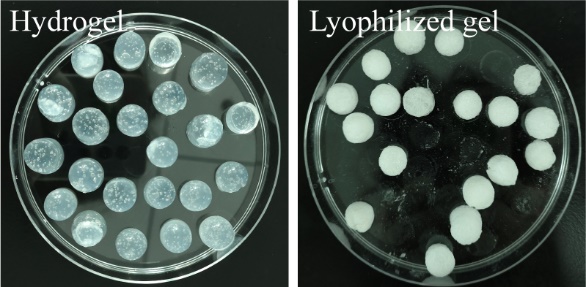


Figure S7: Images of the as-prepared CPO-Alg hydrogels and lyophilized gels.


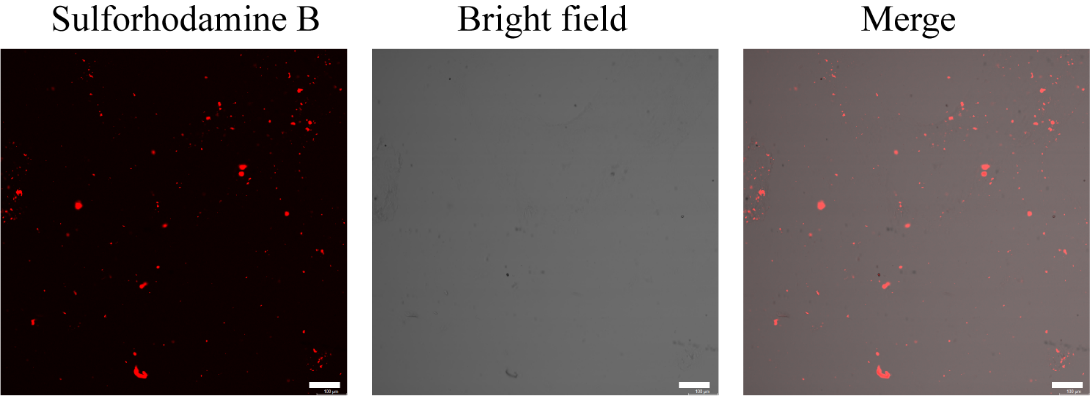


Figure S8: CLSM images of CPO-Alg hydrogel fabricated with sulforhodamine B labelled CPO NPs. Scale bar: 100 μm.


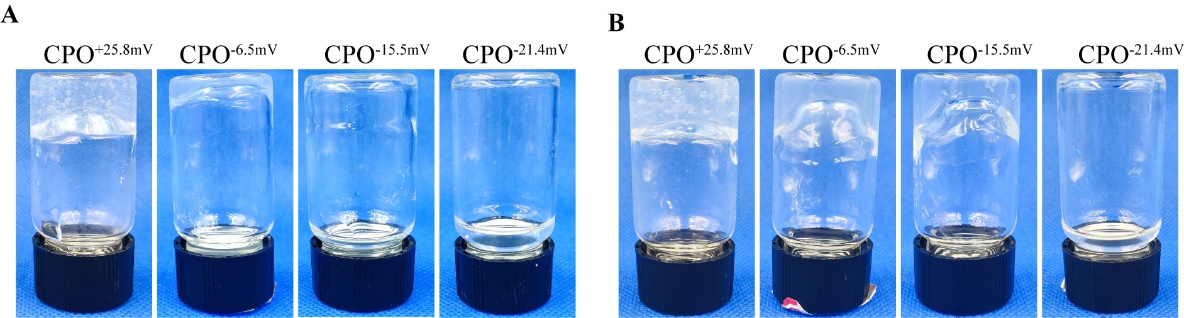


Figure S9: Images of the CPO-Alg gel matrix when the CPO NPs and Alg solution were cross-linked at room temperature for A) 12 h B) 24 h.


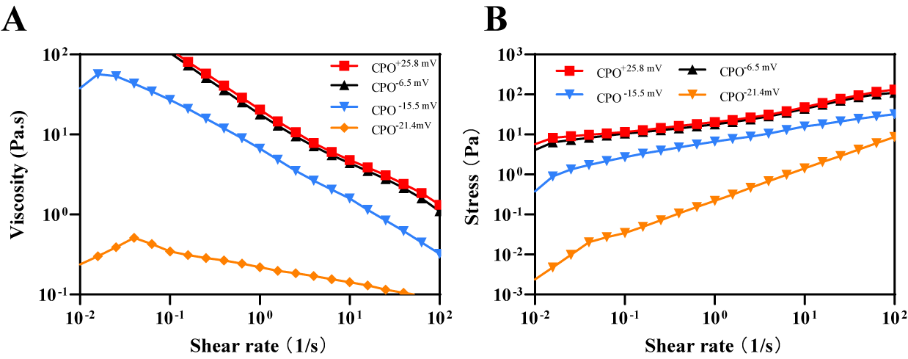


Figure S10: A) Viscosity and B) stress of CPO-Alg gel matrix with different surface charge under different shear rates.


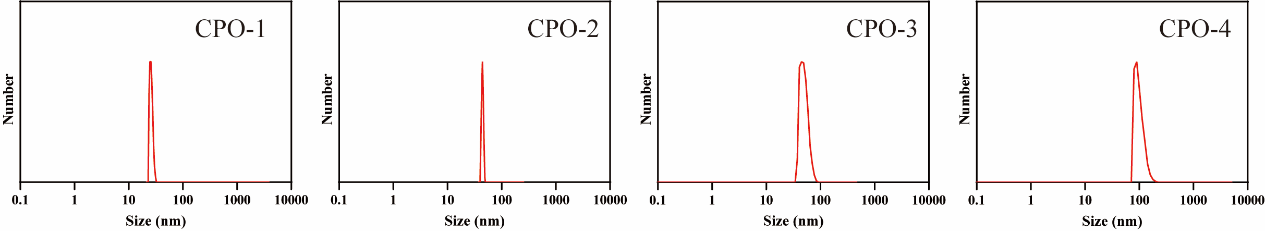


Figure S11. Hydrodynamic size of CPO-1, CPO-2, CPO-3 and CPO-4. The hydrodynamic size of CPO-1, CPO-2, CPO-3 and CPO-4 is 45 nm, 68 nm, 93 nm and 169 nm, respectively.


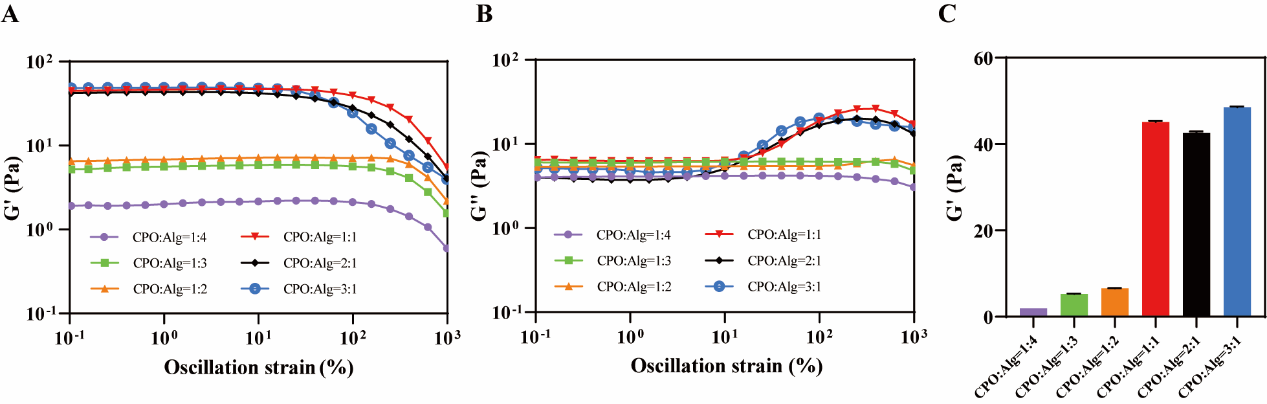


Figure S12: A) G’ of CPO-Alg gel matrix formed by different volume ratio of CPO NPs solutions and Alg solutions in the strain-sweep test. B) G” of CPO-Alg gel matrix formed by different volume ratio of CPO NPs solutions and Alg solutions in the strain-sweep test. C) G’ value of CPO-Alg gel matrix formed by different volume ratio of CPO NPs solutions and Alg solutions at strain of 1% (n=3, mean±SD).


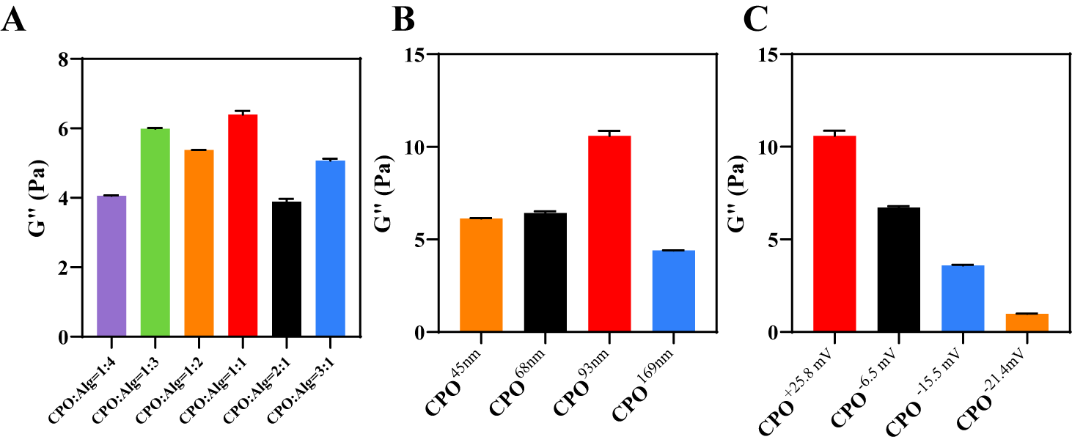


Figure S13: G” values of CPO-Alg gel matrix with different A) volume ratios, B) particle sizes and C) surface charge (n=3, mean±SD).


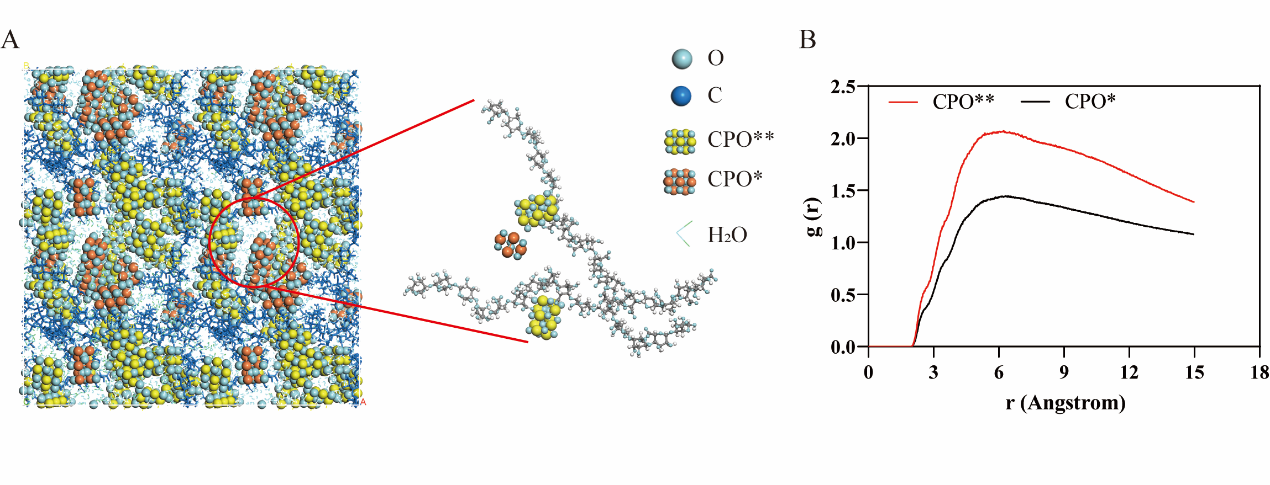


Figure S14. A) Schematic diagram of the CPO-Alg gel. Dark blue chain models indicating Alg molecule, yellow-bule model indicating CPO**, orange-blue model indicating CPO*. B) Radial distribution function (RDF) between CPO NPs in different spatial states and carboxyl oxygen atoms in Alg chain.


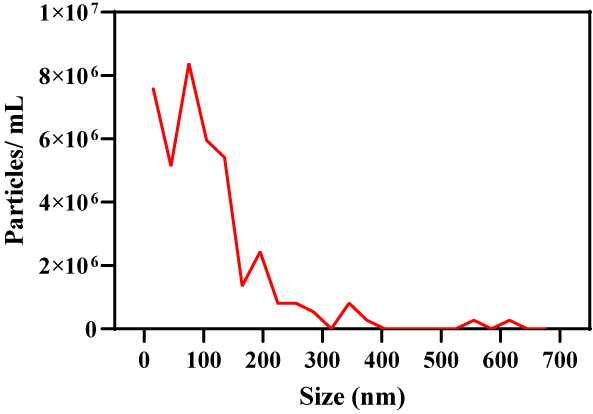


Figure S15: NTA analysis of the solution in the lower chamber after immersion of the gels for 5 h.


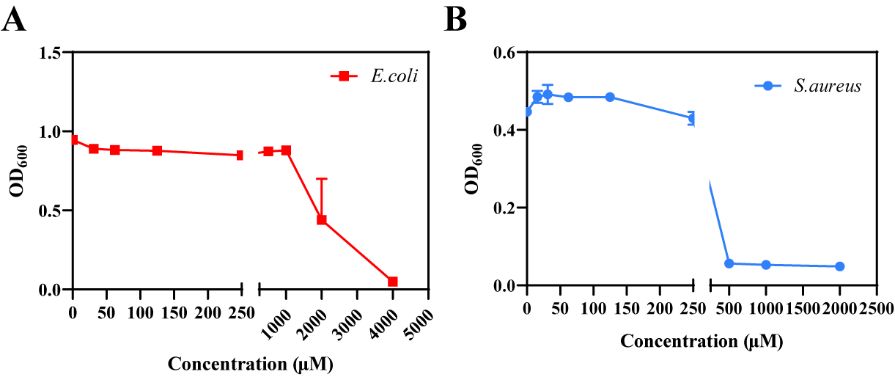


Figure S16: MIC values of CPO NPs for A) *E. coli* and B) *S. aureus* (n=3, mean±SD).


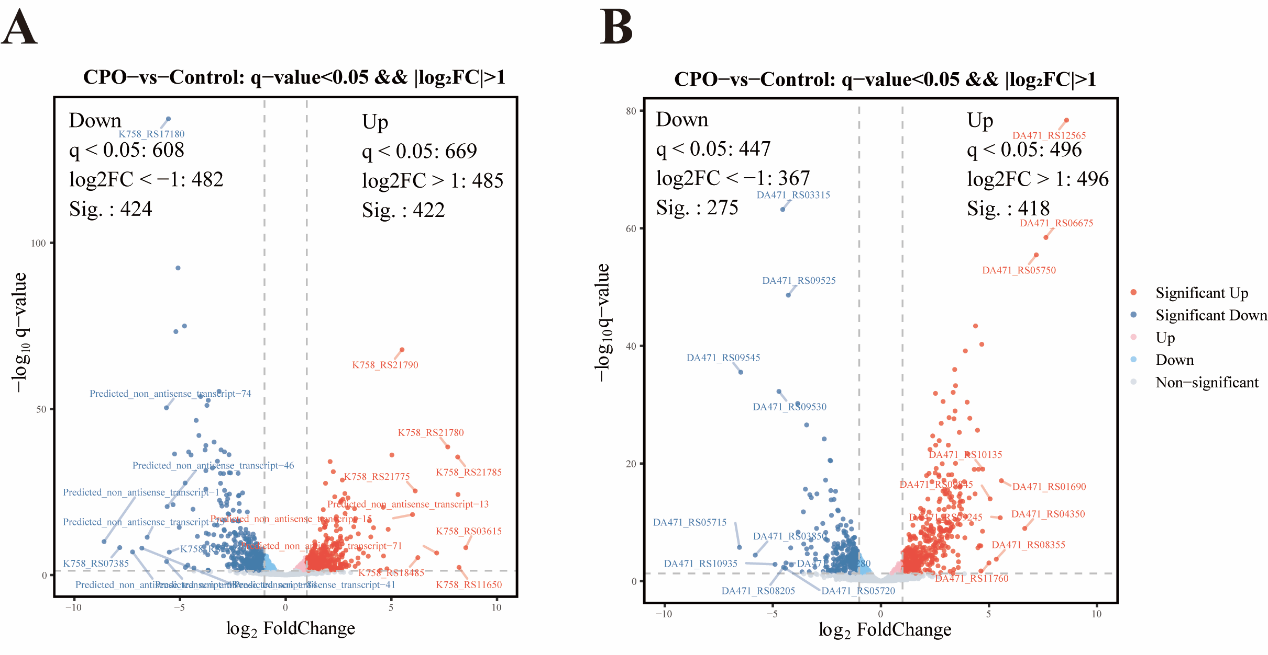


Figure S17: Volcano analysis of DEGs in A) *E. coli* and B) *S. aureus* treated with PBS and CPO NPs.


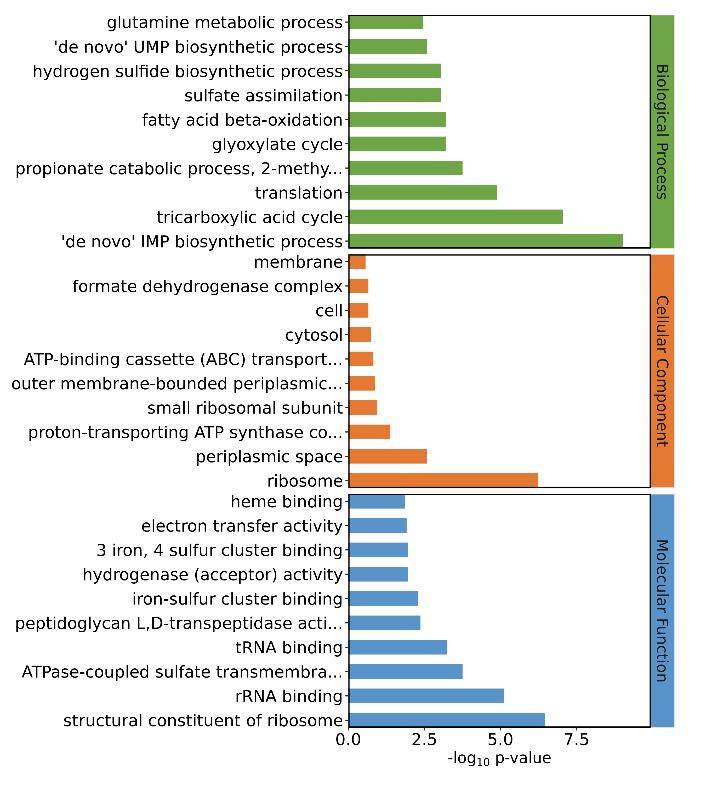


Figure S18: GO annotation analysis of DEGs in *E. coli* treated with PBS and CPO NPs.


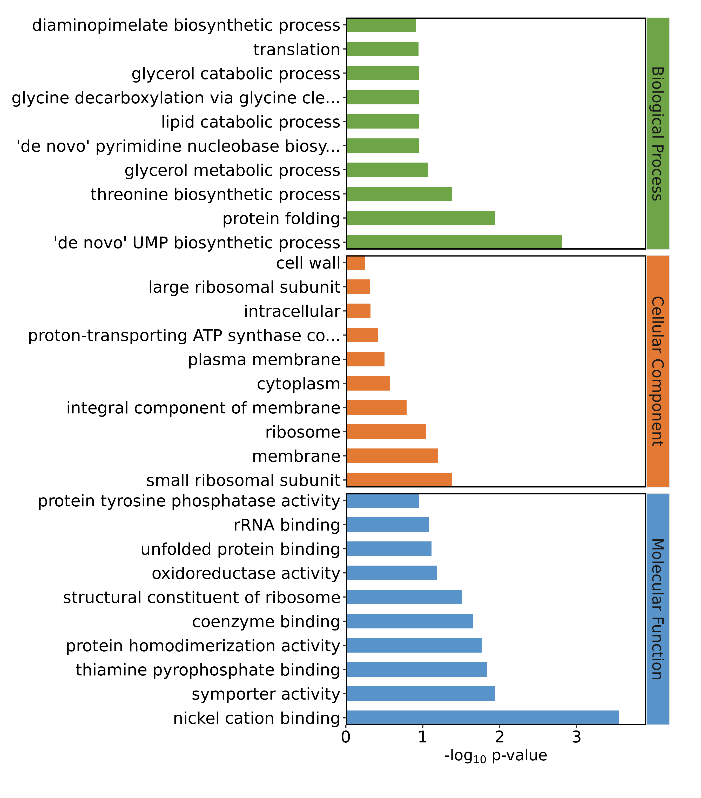


Figure S19: GO annotation analysis of DEGs in *S. aureus* treated with PBS and CPO NPs.

*
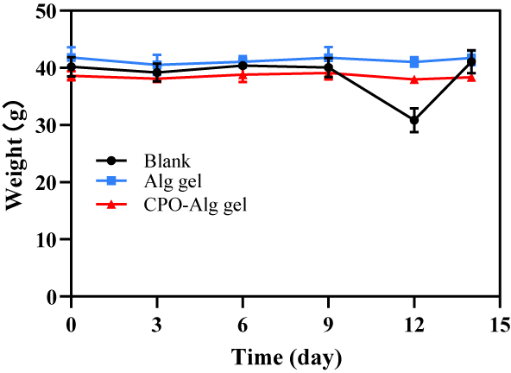
*

Figure S20: Body weight of mice with different treatments (n=5, mean±SD).


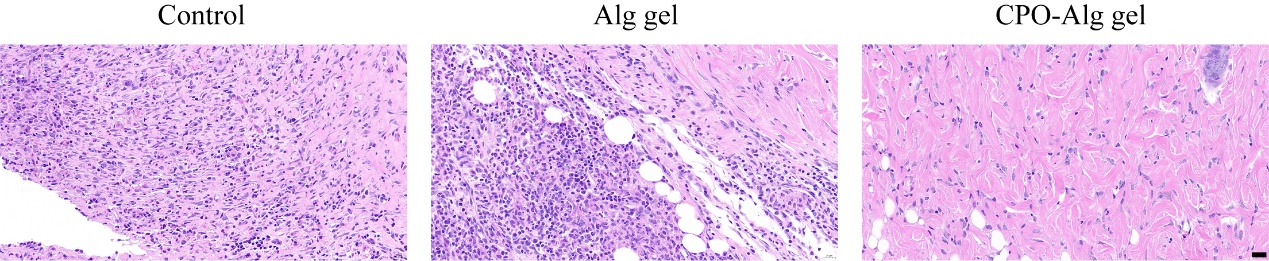


Figure S21: H&E staining images of wounds on day 14 (40 x). Scale bar: 20 μm.


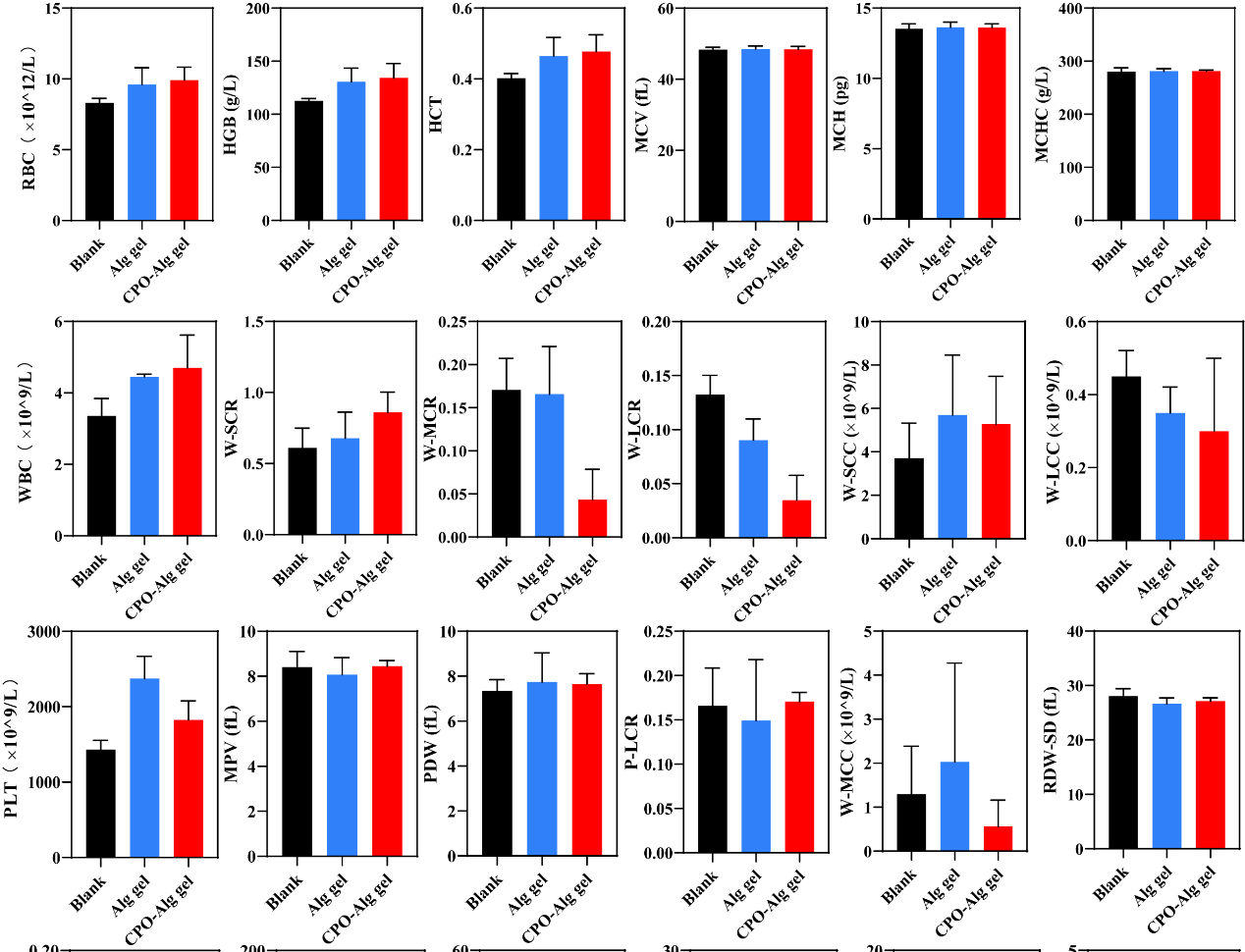


Figure S22: Hematological analysis of Kunming mice with different treatments after 14 days (n=5, mean±SD).


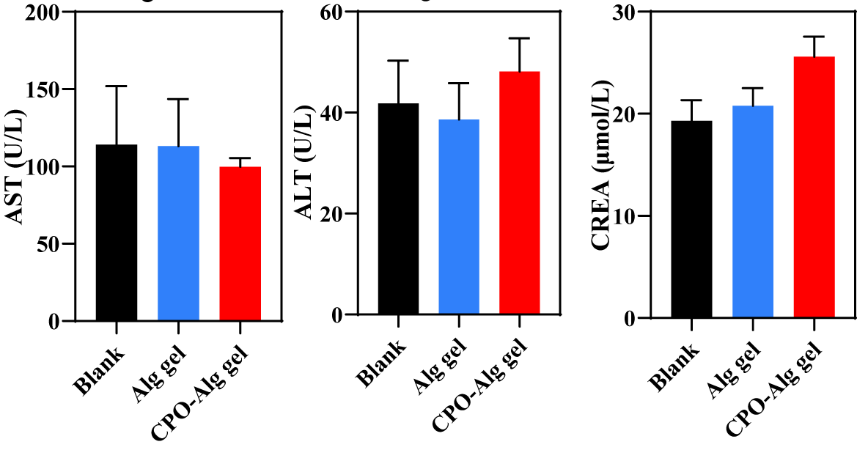


Figure S23: Serum ALT, AST, CREA levels of Kunming mice after different treatments after 14 days (n=5, mean±SD).


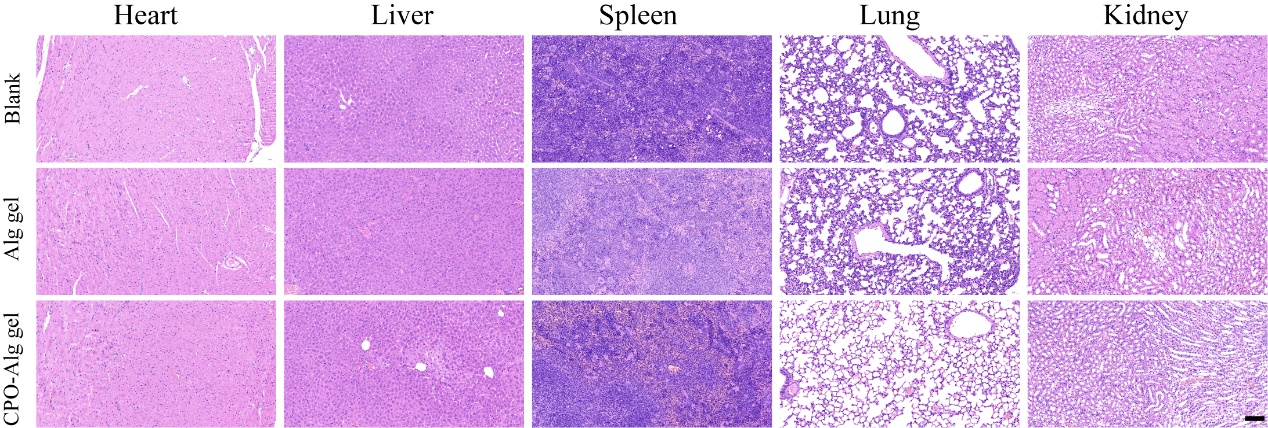


Figure S24: H&E staining images of major organs (heart, liver, spleen, lung and kidney) in different groups after 14 days. Scale bar: 100 μm.

Table S1. MIC assay

| Bacteria | Concentration of CPO NPs (μM) | | | | | | | | MIC(μM) |
| --- | --- | --- | --- | --- | --- | --- | --- | --- | --- |
|  | 31.25 | 62.5 | 125 | 250 | 500 | 1000 | 2000 | 4000 |  |
| *E. coli* | + | + | + | + | + | + | - | - | 2000 |
| *S. aureus* | + | + | + | + | - | - | - | - | 500 |

Positive (+): Indicating growth; Negative (-): Indicating absence of growth.
